# Progesterone Exposure in Pregnancy, Obstetric Stabilization, and Developmental Outcomes: A Focused Review of Direct and Indirect Pathways

**DOI:** 10.3390/children13070953

**Published:** 2026-07-20

**Authors:** Stefan Dugalic, Miroslava Gojnic Dugalic, Milos Milincic, Katarina Ivanovic

**Affiliations:** 1 Clinic for Gynecology and Obstetrics, University Clinical Centre of Serbia, 11000 Belgrade, Serbia; stef.dugalic@gmail.com (S.D.); miroslavagojnicdugalic@yahoo.com (M.G.D.); milosmilincic@gmail.com (M.M.); 2 Faculty of Medicine, University of Belgrade, 11000 Belgrade, Serbia

**Keywords:** progesterone, pregnancy, developmental outcomes, DOHaD, obstetric stabilization, decidua, placenta, preterm birth, confounding by indication, offspring follow-up

## Abstract

**Highlights:**

**What are the main findings?**

- Progesterone-related developmental effects should be interpreted through two distinct frameworks: direct molecular signaling and indirect obstetric effects mediated by maternal–decidual–placental maternal–decidual–placental stabilization, cervical stability, and gestational prolongation.
- Available evidence has not shown a consistent early adverse signal for natural micronized progesterone when used for clearly defined indications, but evidence beyond early childhood remains limited and cannot be generalized to synthetic progestogens or 17-alpha hydroxyprogesterone caproate.

**What are the implications of the main findings?**

- Clinical interpretation should be stratified according to indication, formulation, route, dose, timing, maternal phenotype, and comparator/control group.
- Future studies should report indication-specific and formulation-specific long-term outcomes while explicitly addressing confounding by indication and historical changes in progesterone use.

**Abstract:**

Background: Progesterone is an endogenous pregnancy hormone widely used in reproductive medicine and obstetrics. Its developmental relevance should be interpreted by distinguishing direct molecular plausibility from indirect obstetric effects, including stabilization of the maternal–decidual–placental maternal–decidual–placental environment, cervical stability, and prolongation of gestation. Methods: A focused qualitative narrative review was performed using targeted literature searches and thematic synthesis. This approach was chosen because the available evidence is heterogeneous with respect to indication, formulation, route, dose, timing, comparator group, and offspring follow-up, making quantitative synthesis inappropriate for the present objective. Results: Direct progesterone-related pathways include receptor-mediated signaling, neuroactive metabolites, and biologically plausible epigenetic regulation; however, human evidence for persistent therapy-induced molecular programming remains limited. Indirect pathways are better supported and include decidualization, immune tolerance, placental stabilization, cervical integrity, uterine quiescence, and prevention of prematurity in selected pregnancies. Clinical evidence should be interpreted separately for placebo-controlled trials, untreated pathological cohorts, and observational follow-up studies because underlying maternal conditions may themselves influence offspring outcomes. Potential risk signals also require formulation-specific interpretation, particularly for synthetic progestogens and 17-alpha hydroxyprogesterone caproate, for which long-term observational data and regulatory reassessments have raised concerns regarding efficacy and possible offspring safety signals. Inadvertent exposure to contraceptive progestins during unrecognized pregnancy, including progestin-only preparations and levonorgestrel-releasing intrauterine systems, should be considered separately from therapeutic obstetric progesterone use. Available evidence has not shown a consistent early adverse signal for natural progesterone when used for recognized indications, although long-term offspring data remain limited and cannot be generalized to synthetic progestogens or 17-alpha hydroxyprogesterone caproate. Conclusions: Progesterone-related therapies should be considered indication-specific and formulation-specific rather than interchangeable. Current evidence supports a cautious and comparator-aware interpretation: natural progesterone appears acceptable for recognized clinical indications based on available short-term and early childhood data, but evidence beyond early childhood remains insufficient for broad safety generalizations.

## 1. Introduction

Pregnancy is a biologically plastic period during which fetal development is shaped by maternal nutrition, endocrine status, inflammatory signals, oxygenation, placental transport, and pharmacological exposures. Within the Developmental Origins of Health and Disease (DOHaD) framework, such exposures may influence organ development, endocrine set-points, immune maturation, vascular function, mitochondrial activity, and epigenetic regulation [1,2,3]. However, not every pregnancy exposure acts through direct fetal molecular programming. Some interventions may affect developmental trajectories indirectly by stabilizing the maternal–decidual–placental maternal–decidual–placental environment, reducing inflammation, improving placental function, or prolonging gestation.

Progesterone is a physiological hormone essential for implantation, decidualization, immune tolerance, uterine quiescence, cervical integrity, and maintenance of pregnancy [4,5]. It is widely used in assisted reproduction, threatened miscarriage in selected women, recurrent pregnancy loss in specific clinical contexts, and prevention of preterm birth in women with a short cervix. These indications differ substantially in biological rationale, exposure timing, formulation, route, and expected clinical benefit.

For progesterone, this distinction is particularly important. Progesterone is not a biologically novel exposure, but an endogenous hormone central to implantation, decidualization, immune tolerance, uterine quiescence, cervical integrity, and maintenance of pregnancy [4,5,6]. Exogenous progesterone is usually administered to support pathways that are already physiologically active. Therefore, its potential developmental influence should be framed as a synthesis of possible direct molecular effects and better established indirect obstetric effects rather than as proof of active epigenetic fetal programming.

Available evidence has not shown a consistent early adverse signal for natural progesterone, particularly micronized progesterone used for recognized clinical indications. Nevertheless, long-term follow-up remains incomplete, and evidence cannot be generalized across all progesterone-related formulations. Natural progesterone, synthetic progestogens, and 17-alpha hydroxyprogesterone caproate differ in receptor activity, pharmacokinetics, tissue effects, clinical indications, and safety interpretation [5,6].

The aim of this focused review is to summarize contemporary evidence regarding progesterone exposure in pregnancy and developmental outcomes, while explicitly distinguishing direct molecular mechanisms from indirect maternal–decidual–placental maternal–decidual–placental and obstetric stabilization. Because the primary focus of this review is developmental interpretation of progesterone-related exposure during intended or established pregnancy, assisted reproduction and luteal phase support are considered only when they provide relevant context for early exposure or offspring follow-up. In contrast, accidental exposure during contraceptive failure, including progestin-only pills or levonorgestrel-releasing intrauterine systems, is discussed separately as a boundary issue rather than as a therapeutic obstetric indication. Particular attention is given to clinical indication, formulation, route, dose, timing, comparator group, historical changes in treatment practice, confounding by indication, and long-term offspring follow-up.

## 2. Materials and Methods

### 2.1. Study Design

This article was designed as a focused qualitative narrative review of contemporary evidence. The objective was to identify, organize, and critically synthesize current knowledge regarding progesterone exposure during pregnancy, with emphasis on direct molecular pathways, indirect obstetric effects, and long-term offspring outcomes. The review was not designed to produce pooled treatment estimates, nor to replace a formal systematic review or meta-analysis. Instead, it was developed as a structured thematic synthesis integrating evidence from obstetrics, reproductive medicine, placental biology, immunology, endocrinology, epigenetics, neonatology, pediatrics, and public health.

A qualitative narrative approach was selected because the available literature is methodologically and clinically heterogeneous. Studies differ substantially in progesterone formulation, dose, route, exposure timing, duration, indication, comparator group, and offspring outcome definition. In addition, the clinical use of progesterone-related therapies has changed over time, particularly for prevention of preterm birth and for 17-alpha hydroxyprogesterone caproate. These factors limit the validity of a single quantitative synthesis for the broad developmental question addressed in this review.

### 2.2. Literature Sources and Search Strategy

A targeted, non-systematic literature search was performed using PubMed/MEDLINE, Scopus, Web of Science, and Google Scholar. The final search was performed on 9 July 2026, and publications from January 2015 to June 2026 were prioritized. Earlier landmark studies were also included when they were essential for understanding progesterone physiology, the Developmental Origins of Health and Disease framework, recurrent miscarriage, threatened miscarriage, prevention of preterm birth, regulatory changes, or long-term offspring outcomes.

The search combined terms related to progesterone, micronized progesterone, dydrogesterone, synthetic progestogens, 17-alpha hydroxyprogesterone caproate, pregnancy, miscarriage, short cervix, preterm birth, placenta, decidua, immune tolerance, epigenetics, neurodevelopment, psychiatric outcomes, immune outcomes, childhood outcomes, cancer, contraceptive progestin exposure, progestin-only pills, and levonorgestrel-releasing intrauterine systems. Reference lists of key trials, systematic reviews, regulatory documents, and clinical guidance statements were also screened to identify additional relevant publications.

Because this article was designed as a focused qualitative narrative review rather than a systematic review, no formal protocol was registered, no PRISMA flow diagram was generated, and no formal risk-of-bias or GRADE certainty assessment was performed. Evidence was selected purposively according to relevance to the predefined thematic aims of the review: distinguishing direct molecular plausibility from indirect obstetric stabilization and interpreting offspring outcomes according to formulation, indication, route, dose, timing, comparator group, and confounding by indication.

### 2.3. Thematic Organization and Qualitative Synthesis

The selected literature was organized into thematic domains rather than pooled quantitatively. These domains included: progesterone physiology; decidual and placental mechanisms; direct molecular and epigenetic plausibility; indirect obstetric stabilization; progesterone use in assisted reproduction, miscarriage, and preterm birth prevention; formulation-specific differences between natural progesterone, dydrogesterone, synthetic progestogens, and 17-OHPC; inadvertent contraceptive progestin exposure; and long-term pediatric and developmental outcomes.

The synthesis was narrative and interpretative. Priority was given to placebo-controlled trials, long-term follow-up studies, systematic reviews, meta-analyses, large observational studies, regulatory decisions, and pediatric outcome studies. Observational studies were interpreted cautiously, particularly when the underlying indication for progesterone treatment could itself influence placental function, gestational age at birth, neonatal morbidity, or later child development. No pooled effect estimates were calculated.

### 2.4. Public Health and Translational Perspective

The findings were further interpreted from a translational and public health perspective. Pregnancy was considered a critical window for both immediate obstetric care and long-term disease prevention. The review therefore aimed to evaluate progesterone exposure not only in terms of short-term pregnancy safety and efficacy, but also in terms of its potential implications for offspring growth, neurodevelopment, reproductive health, immune function, metabolic outcomes, and future cardiometabolic risk.

This approach was intended to support individualized counseling and rational pharmacological decision-making in pregnancy. Particular emphasis was placed on the principle that therapeutic use of progesterone should be indication-specific, formulation-specific, dose-aware, historically contextualized, and guided by contemporary evidence, while recognizing that long-term developmental outcomes remain insufficiently studied in many clinical contexts.

## 3. Results and Thematic Synthesis

### 3.1. Direct Molecular Pathways and Indirect Obstetric Pathways

For the purposes of this review, progesterone-related developmental effects are separated into two levels of interpretation. Direct molecular pathways refer to progesterone receptor signaling, non-genomic signaling, neuroactive metabolites, placental gene regulation, and possible epigenetic modulation. Indirect obstetric pathways refer to the ability of progesterone to support implantation, decidualization, immune tolerance, cervical integrity, uterine quiescence, and gestational prolongation.

Direct molecular pathways are biologically plausible because progesterone is central to reproductive tissue function and may influence gene expression through nuclear progesterone receptors and related signaling systems [4,5,6]. Progesterone and its neuroactive metabolites, particularly allopregnanolone, may also interact with the developing nervous system through GABAergic signaling, neuronal maturation, stress regulation, and neuroprotective mechanisms [7,8,9]. However, current human clinical evidence does not establish a consistent pattern of persistent therapy-induced epigenetic or neurodevelopmental programming after standard exposure to natural progesterone.

Indirect obstetric pathways are more strongly supported clinically. During the luteal phase and early pregnancy, progesterone transforms the estrogen-primed endometrium into a receptive tissue, promotes stromal decidualization, regulates extracellular matrix remodeling, supports trophoblast behavior, and contributes to early placental development [10,11,12]. These effects may influence fetal development indirectly by shaping the earliest maternal-fetal environment.

Progesterone also contributes to maternal-fetal immune tolerance. It modulates T-cell responses, supports regulatory immune pathways, influences uterine natural killer cell activity, and limits excessive inflammatory activation at the maternal-fetal interface [13,14,15,16]. This is relevant because maternal and placental inflammation may contribute to placental dysfunction, altered fetal growth, preterm birth, and neurodevelopmental vulnerability.

Later in pregnancy, progesterone contributes to uterine quiescence by regulating contraction-associated proteins, gap junction formation, prostaglandin pathways, calcium signaling, oxytocin responsiveness, and myometrial excitability [17,18]. These mechanisms are obstetric rather than proof of direct fetal programming, but they may influence developmental outcomes when they help prolong intrauterine maturation.

Progesterone also contributes to cervical stability by modulating inflammatory and extracellular matrix pathways. In selected women with a short cervix, vaginal progesterone may reduce the risk of preterm birth [19,20,21,22]. From a developmental perspective, this is best interpreted as an indirect effect, because prematurity itself interrupts fetal organ maturation and exposes the infant prematurely to extrauterine oxygen tension, nutrition, microbial colonization, medical interventions, and environmental stress.

The interpretation of direct and indirect pathways depends on exposure window. Early exposure is primarily related to implantation, decidualization, and placentation, whereas later exposure is more closely related to cervical stability, uterine quiescence, and prevention of preterm birth.

These biologically plausible mechanisms should not be overinterpreted as evidence of active fetal programming in the epigenetic sense. At present, the strongest clinical evidence relates to indirect obstetric stabilization rather than demonstrated persistent changes in offspring DNA expression or long-term disease susceptibility [19,20,21,22].

The developmental interpretation of progesterone also depends on formulation, route, dose, timing, duration, and indication. Natural micronized progesterone, vaginal and oral preparations, dydrogesterone, synthetic progestogens, and 17-alpha hydroxyprogesterone caproate should not be considered biologically interchangeable.

Overall, progesterone exposure in pregnancy should be understood as a framework of direct molecular plausibility and indirect obstetric effects. The latter currently provides the more clinically substantiated pathway linking progesterone therapy with developmental outcomes [19,20,21,22]. These direct and indirect progesterone-related pathways are summarized in Table 1.

### 3.2. Placenta and Decidua as Central Mediators

The decidua and placenta represent the principal biological interface through which progesterone-related signals may influence fetal development. In this context, they should be viewed primarily as mediators of indirect developmental effects: progesterone may support the intrauterine environment in which placental and fetal development occur, without necessarily producing a direct fetal epigenetic effect.

During decidualization, endometrial stromal cells undergo morphological, endocrine, immune, and secretory transformation. The resulting decidual tissue supports implantation, regulates trophoblast invasion, coordinates local immune tolerance, and contributes to early placental anchoring and vascular adaptation [10,11,12]. Impaired decidualization may be associated with early pregnancy loss, abnormal placentation, fetal growth restriction, hypertensive disorders, or preterm birth.

Although the placenta is fetal in origin, its development and function depend on maternal decidual signals, uterine immune cells, vascular remodeling, and endocrine communication. Progesterone contributes to this environment by supporting immune tolerance and limiting excessive inflammatory activation [13,14,15,16]. Through these pathways, it may indirectly influence placental transport, oxygen delivery, endocrine activity, and fetal growth.

Spiral artery remodeling is another important component of the maternal–placental interface. Transformation of high-resistance maternal vessels into low-resistance uteroplacental channels is necessary for adequate placental perfusion. Disruption of decidual, immune, or vascular pathways may result in placental insufficiency and altered fetal exposure to oxygen, nutrients, hormones, and inflammatory mediators.

Placental and decidual development are also regulated by epigenetic mechanisms, including DNA methylation, histone modifications, chromatin remodeling, and non-coding RNAs. Although these mechanisms are central to developmental biology, direct clinical evidence linking therapeutic progesterone exposure to specific persistent placental epigenetic changes remains limited [3,12]. Therefore, epigenetic plausibility should be distinguished from demonstrated epigenetic modulation in human offspring.

Overall, the placenta and decidua should be considered central mediators of indirect progesterone-related developmental effects. Progesterone may support implantation, placental development, immune tolerance, vascular adaptation, and regulated maternal-fetal exchange. However, most clinical studies have focused on live birth, gestational age, neonatal morbidity, or early childhood follow-up rather than placental biomarkers, molecular endpoints, or long-term offspring health. The principal decidual and placental pathways linking progesterone with indirect developmental effects are summarized in Table 2.

### 3.3. Clinical Evidence, Indications, Comparator Structures, and Offspring Outcomes

Progesterone is used in several reproductive and obstetric settings, but its clinical and developmental meaning differs according to indication, timing, formulation, route, dose, duration, maternal phenotype, and comparator group. Consequently, progesterone exposure should not be interpreted as a single uniform intervention.

For interpretation of clinical evidence, comparator structure is critical. Placebo-controlled randomized trials are most informative for isolating the effect of progesterone within a defined indication. Untreated pathological cohorts are more difficult to interpret because the underlying condition that led to progesterone use may itself influence placental function, pregnancy duration, and child development. Observational studies and registry-based studies require careful attention to confounding by indication and residual confounding.

#### 3.3.1. Assisted Reproduction and Luteal Phase Support

Progesterone is routinely used for luteal phase support in assisted reproductive technology, primarily to compensate for altered corpus luteum function associated with ovarian stimulation and follicular aspiration. In the context of the present review, ART and luteal phase support are not treated as a separate therapeutic focus, but as an early-exposure context relevant to implantation, decidualization, and early placentation [23,24].

Interpretation of offspring outcomes after ART-related progesterone exposure is particularly challenging. Comparator groups often involve different routes, formulations, or durations of luteal support rather than true untreated controls. In addition, infertility diagnosis, parental characteristics, ovarian stimulation, embryo culture, cryopreservation, embryo transfer practices, multiple pregnancy risk, and placental development may influence offspring outcomes independently of progesterone. Therefore, available ART-related evidence cannot reliably isolate the independent developmental contribution of progesterone and should not be generalized to obstetric indications such as threatened miscarriage, recurrent pregnancy loss, or short cervix [25,26].

#### 3.3.2. Recurrent Pregnancy Loss and Threatened Miscarriage

The PROMISE trial was a placebo-controlled randomized trial and did not demonstrate a significant live birth benefit from vaginal micronized progesterone in the overall population of women with unexplained recurrent miscarriage [25]. Because the comparator was placebo within a defined clinical phenotype, this trial is informative for treatment efficacy, but it was not primarily designed to evaluate placental molecular endpoints or long-term offspring development.

The PRISM trial was also placebo-controlled and found no statistically significant overall increase in live birth among women with early pregnancy bleeding. However, subgroup analyses suggested greater benefit in women who had both current bleeding and a history of previous miscarriage, particularly those with multiple previous losses [26,27]. Meta-analytic evidence and current guidance therefore support selective rather than universal administration of progesterone in early pregnancy [28,29,30].

These early-pregnancy trials provide reassurance regarding short-term exposure to vaginal micronized progesterone, but their main endpoints were live birth and immediate safety. They provide limited information on placental biology, epigenetic outcomes, or long-term child development. Therefore, they should not be used alone to make broad claims about developmental neutrality.

#### 3.3.3. Short Cervix and Prevention of Preterm Birth

Vaginal progesterone may reduce preterm birth and adverse perinatal outcomes in selected singleton pregnancies with a mid-trimester short cervix [19,20,21,22]. In this indication, the strongest developmental rationale is indirect: progesterone may prolong gestation and thereby reduce prematurity-related developmental exposures.

Preterm birth interrupts intrauterine maturation of the brain, lungs, intestine, immune system, endocrine axes, kidneys, and metabolic tissues. By prolonging gestation, progesterone may reduce exposure to prematurity-related developmental risks even if it does not exert a direct programming effect on fetal organs.

Available early childhood follow-up is generally reassuring. No clear differences in neurodevelopmental, behavioral, physical, or general health outcomes were observed after antenatal vaginal progesterone exposure for short cervix at approximately two years of age [20,31]. Nevertheless, these data mainly address early childhood and cannot exclude later-emerging outcomes.

A systematic review of five randomized controlled trials, comprising seven publications and 4222 child outcome measurements from 6 months to 8 years of age, found no evidence of benefit or harm after prenatal progesterone treatment for prevention of preterm birth [32]. However, the available studies predominantly evaluated exposure during the second or third trimester, and evidence regarding first-trimester exposure, adolescence, reproductive outcomes, and adult cardiometabolic health remained insufficient.

More recently, a retrospective observational study reported lower language and personal–social developmental scores among children with prenatal progesterone exposure, while gross motor, fine motor, and adaptive behavior scores did not differ significantly [33]. These findings should be interpreted cautiously because observational designs cannot fully separate medication exposure from the underlying maternal indication, such as bleeding, infertility, miscarriage risk, cervical shortening, inflammatory vulnerability, or threatened preterm birth. The study reinforces the need for prospective, indication-specific, formulation-specific, and comparator-aware long-term follow-up.

#### 3.3.4. Synthetic Progestogens, 17-Alpha Hydroxyprogesterone Caproate, and Historical Changes in Practice

The regulatory history of 17-alpha hydroxyprogesterone caproate (17-OHPC) demonstrates the importance of formulation-specific and historically contextualized evaluation. The original NICHD Maternal-Fetal Medicine Units Network trial by Meis et al. reported that weekly intramuscular 250 mg 17-OHPC, started between 16 and 20 weeks of gestation and continued until delivery or 36 weeks, reduced recurrent preterm birth among women with a prior spontaneous preterm delivery [34]. This trial supported the subsequent clinical adoption of 17-OHPC for prevention of recurrent preterm birth. However, the later PROLONG trial, a larger multicenter international randomized double-blind confirmatory study, did not demonstrate a reduction in recurrent preterm birth before 35 weeks or in neonatal morbidity among women with a prior singleton spontaneous preterm birth [35]. The FDA withdrawal of approval and European regulatory reassessment were therefore based primarily on lack of confirmed efficacy, together with uncertainty regarding long-term safety [36,37].

These regulatory decisions should not be automatically extrapolated to natural vaginal progesterone. Natural progesterone, dydrogesterone, and synthetic progestogens differ in molecular structure, receptor activity, metabolism, pharmacokinetics, placental exposure, tissue effects, clinical indications, and dose schedules. Historical mixing of these agents under the broad label of “progestogens” may reduce data consistency and may obscure formulation-specific developmental interpretation.

#### 3.3.5. Dydrogesterone and Fetal Safety Signals

Dydrogesterone should also be interpreted separately from natural micronized progesterone and from 17-OHPC. It is an orally active retroprogesterone with pharmacological and clinical properties that differ from vaginal micronized progesterone. Its use has been evaluated mainly in the context of luteal phase support and early pregnancy, but pediatric and developmental data remain less extensive than obstetric efficacy data.

A retrospective case–control study by Zaqout et al. reported an association between first-trimester dydrogesterone exposure and congenital heart disease in offspring [38]. This signal is clinically relevant because congenital heart defects represent an important pediatric outcome and because exposure occurs during early cardiac development. However, the finding should be interpreted cautiously because the study was observational and could not fully exclude recall bias, residual confounding, or confounding by indication.

Other evidence has not consistently confirmed this association. In a large phase III randomized trial comparing oral dydrogesterone with micronized vaginal progesterone for luteal phase support in IVF, oral dydrogesterone was non-inferior for ongoing pregnancy at 12 weeks and had a similar safety profile to micronized vaginal progesterone [39]. More recent systematic review and meta-analytic evidence has also not shown dydrogesterone to be an additional risk factor for congenital anomalies when used in the first trimester for threatened or recurrent pregnancy loss or luteal support, although certainty remains limited by the fact that congenital anomalies were often secondary outcomes rather than primary endpoints [40].

Therefore, dydrogesterone should be discussed as a formulation-specific exposure with mixed but clinically important safety literature. Current evidence does not justify grouping it together with either natural vaginal progesterone or 17-OHPC, and further prospective pediatric follow-up remains necessary.

#### 3.3.6. Potential Risk Signals: 17-Alpha Hydroxyprogesterone Caproate and Cancer-Related Concerns

Potential risk signals should be interpreted separately for each progesterone-related formulation. This is particularly important for 17-alpha hydroxyprogesterone caproate, which differs from natural progesterone in molecular structure, pharmacokinetics, metabolism, duration of exposure, receptor activity, tissue effects, and regulatory history. Therefore, findings related to 17-OHPC should not be extrapolated to vaginal micronized progesterone or other natural progesterone preparations.

Long-term observational evidence has raised concern regarding a possible association between in utero exposure to 17-OHPC and later cancer risk in offspring. In the Child Health and Development Studies cohort, in utero exposure to 17-OHPC was associated with an increased risk of cancer among offspring, particularly when exposure occurred in early pregnancy [41]. However, these data should be interpreted cautiously because exposure occurred in a historical treatment era, indications differed from current practice, and residual confounding cannot be excluded.

Cancer-related outcomes are methodologically difficult to evaluate because they require large exposed populations, long latency periods, reliable exposure ascertainment, and linkage to cancer registries. Confounding by indication may also be relevant, as women treated with 17-OHPC may differ from untreated women with respect to previous pregnancy loss, threatened abortion, cervical insufficiency, inflammation, comorbidities, socioeconomic characteristics, and patterns of obstetric surveillance.

Regulatory reassessments of 17-OHPC have emphasized two distinct issues: lack of confirmed efficacy for prevention of recurrent preterm birth and uncertainty regarding long-term safety [36,37]. The possible cancer-related signal should therefore be regarded as formulation-specific and hypothesis-generating rather than as definitive evidence of carcinogenicity. Nevertheless, it reinforces the need for formulation-specific, dose-aware, and era-specific long-term follow-up of children exposed to progesterone-related therapies in utero.

#### 3.3.7. Inadvertent Exposure to Progestin-Only Contraception and Levonorgestrel-Releasing Intrauterine Systems

Inadvertent exposure to contraceptive progestins during an unrecognized pregnancy represents a different clinical scenario from therapeutic progesterone use in obstetrics. Progestin-only pills, emergency contraception, implants, injectable contraceptives, and levonorgestrel-releasing intrauterine systems are used to prevent pregnancy, not to stabilize an established pregnancy. Therefore, their dose, route, timing, pharmacology, indication, and comparator structure differ substantially from progesterone therapy used for luteal support, threatened miscarriage, recurrent pregnancy loss, or short cervix.

For oral contraceptive exposure around conception or during early unrecognized pregnancy, large registry-based data have generally not shown a major increase in congenital anomalies [42]. However, more recent cohort data suggest that periconceptional oral contraceptive exposure may be associated with some adverse pregnancy outcomes, including preterm birth and low birthweight, although these findings may depend on timing, estrogen dose, and progestin type [43]. Evidence specific to progestin-only pills remains less extensive than evidence for oral contraceptives overall, and available data mainly address congenital anomalies and early pregnancy outcomes rather than long-term neurodevelopmental, psychiatric, immune, reproductive, metabolic, or cancer-related endpoints.

Levonorgestrel-only emergency contraception failure has not been associated with an increased risk of major congenital malformations or adverse neonatal outcomes in prospective cohort data [44]. Nevertheless, this evidence should not be directly generalized to all progestin-only contraceptive preparations, because dose, timing, route, and pharmacokinetics differ.

Pregnancy with a levonorgestrel-releasing intrauterine system in situ requires separate interpretation. The main clinical concern is not proven direct fetal programming by levonorgestrel, but the pregnancy context itself, including the higher relative probability of ectopic pregnancy among contraceptive failures and increased risks of miscarriage, infection, and preterm birth when an intrauterine device remains in place. A systematic review of pregnancies with an IUD in situ found increased risks of spontaneous abortion, preterm delivery, septic abortion, and chorioamnionitis, while early IUD removal appeared to improve outcomes but did not eliminate risk [45]. Long-term offspring data after levonorgestrel-IUS exposure in continuing pregnancies remain limited, and available evidence is insufficient to draw conclusions about later neuropsychiatric, immune, reproductive, metabolic, or cancer outcomes.

Thus, inadvertent contraceptive progestin exposure should be presented as a boundary issue rather than as part of the core therapeutic progesterone literature. It supports the broader conclusion that pregnancy progestogen exposure must be interpreted according to formulation, dose, route, indication, timing, and comparator group.

#### 3.3.8. Neuropsychiatric, Neurological, and Immune Outcomes in Offspring

The most clinically relevant long-term offspring domains include neurodevelopmental, psychiatric, neurological, immune, reproductive, metabolic, and cardiometabolic outcomes. However, available evidence remains uneven across these domains. Most follow-up studies after antenatal progesterone exposure have focused on early childhood development, physical growth, general health, and broad behavioral outcomes, while later psychiatric diagnoses, school-age cognitive function, immune-mediated disease, pubertal development, reproductive health, and adult cardiometabolic outcomes remain insufficiently studied.

Neurodevelopmental and neurological outcomes are biologically plausible endpoints because progesterone and its neuroactive metabolites, particularly allopregnanolone, participate in fetal brain maturation, GABAergic signaling, neuroprotection, stress regulation, and neuronal development. Nevertheless, biological plausibility does not establish persistent therapy-induced neurodevelopmental programming in humans. A systematic review of randomized follow-up studies, including children aged from 6 months to 8 years, found no evidence of benefit or harm after prenatal progesterone treatment for prevention of preterm birth, but emphasized that evidence for first-trimester exposure and outcomes beyond early childhood remains limited [32].

More recent observational data have suggested lower language and personal–social developmental scores among children exposed to maternal progesterone during pregnancy, while gross motor, fine motor, and adaptive behavior scores did not differ significantly [33]. These findings should be interpreted cautiously because observational designs cannot fully separate progesterone exposure from the underlying maternal indication, including infertility, assisted reproduction, early bleeding, miscarriage risk, cervical shortening, threatened preterm birth, inflammation, maternal stress, or prematurity.

Psychiatric outcomes are less clearly defined. Few studies have been designed to evaluate later psychiatric diagnoses such as anxiety disorders, depressive disorders, attention-deficit/hyperactivity disorder, autism spectrum disorder, or other behavioral and emotional outcomes after therapeutic natural progesterone exposure in pregnancy. Some data on synthetic progestins have raised concern regarding psychiatric or neurodevelopmental outcomes, but these findings are limited by study design, exposure heterogeneity, and potential selection or reporting bias [46]. Therefore, current evidence is insufficient to conclude either long-term psychiatric safety or long-term psychiatric risk.

Immune outcomes also deserve attention because progesterone influences maternal-fetal immune tolerance, decidual immune cell function, inflammatory signaling, and the balance between tolerance and immune activation at the maternal-fetal interface. These pathways may theoretically influence fetal immune maturation and later susceptibility to atopy, asthma, autoimmune disease, or infection-related morbidity. However, direct human evidence linking therapeutic progesterone exposure to persistent immune programming in offspring is limited. Most studies were not designed to evaluate immune phenotypes, immunological biomarkers, allergic disease, or autoimmune outcomes as primary endpoints.

Overall, current evidence does not demonstrate a consistent adverse signal for neurodevelopmental, psychiatric, neurological, or immune outcomes after natural progesterone used for recognized indications. However, the absence of a consistent early signal should not be interpreted as definitive long-term safety. Future studies should include standardized neurodevelopmental testing, validated psychiatric and behavioral assessments, immune and allergic outcomes, sex-specific analyses, and follow-up into adolescence and adulthood.

#### 3.3.9. Integrated Interpretation by Comparator Group, Risk Domain, and Confounding Structure

The interpretation of progesterone-related exposure depends not only on comparator group and indication, but also on the risk domain being evaluated. Short-term obstetric outcomes, congenital anomalies, early childhood neurodevelopment, psychiatric outcomes, immune-mediated disease, reproductive development, cardiometabolic outcomes, and cancer risk require different follow-up periods, sample sizes, and methods of ascertainment. Rare or delayed outcomes, such as malignancy or adolescent psychiatric diagnoses, cannot be adequately excluded by trials designed primarily for live birth, preterm birth, or neonatal morbidity.

Current evidence supports an indication-specific, formulation-specific, timing-specific, and comparator-aware interpretation. PROMISE and PRISM were placebo-controlled trials and do not support universal progesterone administration in women with recurrent miscarriage or early pregnancy bleeding, although they suggest possible benefit in selected clinical phenotypes [25,26,27,28,29,30]. In women with a short cervix, the principal developmental benefit is likely mediated through prevention of prematurity [19,20,21,22].

A major limitation of the literature is confounding by indication. Women receiving progesterone may have infertility, assisted reproduction, previous pregnancy loss, early bleeding, cervical shortening, previous preterm birth, inflammatory conditions, or placental vulnerability. These factors may themselves influence placental function, pregnancy duration, neonatal morbidity, and offspring neurodevelopment. Observational studies are therefore most useful when they adjust for the underlying indication and relevant obstetric risk factors, but residual confounding often remains possible.

Available evidence for natural progesterone is broadly reassuring when used for recognized indications, particularly for short-term and early childhood outcomes. However, the absence of a clear early adverse signal should not be interpreted as definitive evidence of long-term developmental neutrality, especially for outcomes that may emerge in adolescence or adulthood. Key clinical and offspring outcome studies relevant to progesterone-related exposure in pregnancy are summarized in Table 3.

## 4. Discussion: Clinical and Public Health Implications, Limitations, and Future Research

Progesterone therapy should be individualized according to indication, formulation, route, timing, dose, and maternal phenotype. Counseling should distinguish natural progesterone from synthetic progestogens and should address both the expected obstetric benefit and the limitations of available long-term safety evidence. The comparator structure of the evidence should also be explained: conclusions drawn from placebo-controlled trials are not equivalent to conclusions drawn from untreated pathological cohorts or observational follow-up studies.

From a pediatric perspective, the central issue is not only whether progesterone-related therapies improve obstetric endpoints, but whether they influence developmental trajectories after birth. Relevant child-centered outcomes include survival without major neonatal morbidity, brain maturation, language development, behavior, school-age cognition, psychiatric diagnoses, immune-mediated disease, growth, pubertal development, reproductive health, and later cardiometabolic risk. These outcomes require longer follow-up than most obstetric trials provide. Trials designed primarily for live birth, preterm birth, or neonatal morbidity may be insufficient to evaluate later-emerging pediatric outcomes, particularly those that appear in school age, adolescence, or adulthood.

A developmental-outcomes perspective broadens the assessment of safety beyond congenital anomalies and immediate neonatal outcomes. Relevant long-term domains include neurodevelopment, behavior, growth, body composition, metabolic health, immune function, pubertal development, reproductive function, and cardiometabolic risk. However, at present, available human evidence is insufficient to claim that therapeutic progesterone induces or prevents long-term epigenetic fetal programming in a definitive sense.

At the same time, the potential risks of treatment must be interpreted against the risks of the underlying pregnancy complication. Miscarriage, placental dysfunction, inflammation, cervical shortening, and preterm birth may themselves influence fetal development. In selected women, progesterone may reduce these adverse exposures by stabilizing early pregnancy or prolonging gestation. This creates a central methodological challenge: offspring outcomes may reflect the treated condition, the treatment, or both.

Future studies should separate progesterone formulations and gestational exposure windows, account for confounding by indication, and report outcomes according to fetal sex. They should also predefine comparator groups, distinguish placebo-controlled evidence from untreated pathological cohorts, and document route, dose, treatment duration, and historical era of care. Placental histology, vascular and inflammatory biomarkers, transcriptomics, DNA methylation, and non-coding RNAs may help clarify whether progesterone-related changes at the maternal-fetal interface contribute to developmental outcomes.

Large pregnancy medication registries and linked maternal-child datasets are needed to identify rare or delayed outcomes. These systems should integrate maternal indication, formulation, dose, route, timing, pregnancy outcome, placental phenotype, neonatal outcome, and long-term child health. Harmonized reporting would reduce the inconsistency created by historical modifications in clinical indications and therapeutic regimens.

This review has several limitations. Its focused and qualitative design was intended to provide an integrated clinical and developmental interpretation rather than an exhaustive systematic assessment of all available evidence. No formal risk-of-bias assessment, certainty-of-evidence grading, or quantitative synthesis was performed. Interpretation was also limited by heterogeneity in progesterone formulations, routes, doses, treatment indications, gestational timing, historical treatment practices, comparator groups, and offspring outcome definitions. Confounding by indication remains particularly important because the maternal conditions leading to progesterone treatment may themselves influence placental function, pregnancy duration, and child development. A further limitation is that different long-term risk domains are not equally represented in the literature. Neurodevelopmental and general health outcomes have been assessed more often than psychiatric diagnoses, immune-mediated disease, reproductive outcomes, cardiometabolic outcomes, or cancer. In addition, inadvertent contraceptive progestin exposure during unrecognized pregnancy and therapeutic progesterone administration for obstetric indications should not be analyzed as equivalent exposures. These scenarios differ in indication, dose, route, formulation, timing, and comparator structure. The available evidence is therefore insufficient to support broad conclusions across all progestogen exposures in pregnancy. Although observational studies were interpreted in relation to whether they addressed this problem, residual confounding cannot be excluded. The principal strength of this review is the integration of mechanistic, placental, clinical, regulatory, and long-term offspring evidence while explicitly separating direct molecular plausibility from indirect obstetric stabilization. The principal evidence gaps and future research priorities are summarized in Table 4.

## 5. Conclusions

Progesterone is an essential pregnancy hormone with central roles in implantation, decidualization, immune tolerance, placental development, uterine quiescence, cervical stability, and maintenance of gestation. Its developmental relevance should be interpreted through two distinct but related frameworks: direct molecular plausibility and indirect obstetric stabilization. Current evidence more strongly supports the indirect pathway, particularly through support of the maternal–decidual–placental maternal–decidual–placental environment and prevention of prematurity in selected pregnancies.

Clinical benefit remains indication-dependent. Progesterone is established for luteal phase support, may benefit selected women with early pregnancy bleeding and previous miscarriage, and may reduce preterm birth in singleton pregnancies with a short cervix. However, these effects should primarily be interpreted within their specific clinical contexts and comparator structures, rather than as evidence of generalized fetal programming or universal developmental benefit.

Progesterone-related therapies should not be considered interchangeable. Natural progesterone, dydrogesterone, synthetic progestogens, contraceptive progestins, and 17-OHPC differ in pharmacology, clinical indication, receptor activity, regulatory history, dose regimens, route of administration, and safety interpretation. Conclusions regarding one formulation, route, indication, or historical era should therefore not be generalized to others. In particular, the possible cancer-related signal described for in utero 17-OHPC exposure should be interpreted as formulation-specific and hypothesis-generating, and should not be extrapolated to natural vaginal progesterone.

Long-term offspring evidence remains limited, particularly beyond early childhood. Current data are insufficient to support broad safety generalizations regarding later neurodevelopmental, psychiatric, neurological, immune, reproductive, metabolic, or cardiovascular outcomes. Evidence on inadvertent contraceptive progestin exposure, including progestin-only preparations and levonorgestrel-releasing intrauterine systems, is also limited and should be considered separately from therapeutic obstetric progesterone use. Until more robust long-term evidence becomes available, progesterone-related therapies should be used rationally, conservatively, and according to clearly defined clinical indications, with interpretation stratified by formulation, dose, route, timing, comparator group, and maternal indication.

## Figures and Tables

**Table 1:** Direct and indirect progesterone-related pathways relevant to developmental outcomes.

Pathway Type	Examples	Developmental Interpretation
Direct molecular plausibility	Progesterone receptor signaling, non-genomic signaling, neuroactive metabolites, possible epigenetic regulation	Biologically plausible, but persistent therapy-induced programming in humans remains insufficiently demonstrated
Early indirect obstetric effects	Endometrial receptivity, implantation, decidualization, trophoblast regulation, immune tolerance	May stabilize the early maternal-fetal interface and reduce adverse inflammatory or placental exposures
Placental-decidual stabilization	Regulated maternal-fetal exchange, vascular adaptation, limitation of excessive inflammation	May influence fetal growth and maturation indirectly through placental function
Later indirect obstetric effects	Cervical stability, uterine quiescence, prevention of preterm birth in selected pregnancies	May reduce prematurity-related developmental risks by prolonging intrauterine maturation
Formulation, dose, and timing	Natural progesterone, dydrogesterone, synthetic progestogens, 17-OHPC; first vs. second/third trimester exposure	Prevents inappropriate generalization across therapies and historical treatment eras

**Table 2:** Decidual and placental pathways linking progesterone with indirect developmental effects.

Pathway	Progesterone-Related Effect	Indirect Developmental Relevance
Decidual transformation	Supports stromal differentiation and secretory function	Promotes stable implantation and early placentation
Trophoblast regulation	Acts through decidual and immune pathways	May influence placental anchoring and maternal-fetal exchange
Immune tolerance	Limits excessive inflammatory activation	May reduce inflammation-related developmental stressors
Vascular adaptation	Supports an environment permissive for spiral artery remodeling	May influence placental perfusion and fetal growth
Placental transport and endocrine function	Supports pregnancy-maintaining endocrine and metabolic exchange	May affect fetal maturation and growth indirectly
Epigenetic regulation	Placental and decidual gene regulation is biologically plausible	Human evidence for persistent progesterone-induced epigenetic modulation remains limited

**Table 3:** Key clinical and offspring outcome studies relevant to progesterone-related exposure in pregnancy.

Study	Design and Population	Treatment/Exposure	Main Endpoint	Main Result	Developmental Interpretation
PROMISE	Placebo-controlled RCT; women with unexplained recurrent miscarriage	Vaginal micronized progesterone vs. placebo	Live birth	No significant overall live birth benefit	Informative for early pregnancy efficacy, but not designed for long-term child outcomes
PRISM	Placebo-controlled RCT; women with early pregnancy bleeding	Vaginal micronized progesterone vs. placebo	Live birth	No significant overall benefit; possible benefit in women with bleeding and previous miscarriage	Supports targeted rather than universal use; limited developmental follow-up
OPPTIMUM	Placebo-controlled RCT; women at risk of preterm birth	Vaginal progesterone vs. placebo	Fetal death or birth < 34 weeks; neonatal and childhood outcomes	No clear improvement in primary obstetric/neonatal outcomes; early childhood follow-up generally reassuring	Important because it includes child follow-up, but later outcomes remain insufficient
EPPPIC	Individual participant data meta-analysis of randomized trials	Vaginal progesterone, 17-OHPC, or oral progesterone	Preterm birth and neonatal outcomes	Benefit most relevant in high-risk singleton pregnancies, especially short cervix; no support for unselected multifetal use	Demonstrates indication-specific benefit and need to separate formulations
Meis et al.	Placebo-controlled RCT; women with prior spontaneous preterm birth	Weekly intramuscular 17-OHPC 250 mg vs. placebo	Recurrent preterm birth	Reduced preterm birth < 37, <35, and <32 weeks	Original positive evidence supporting 17-OHPC use; pediatric benefit inferred through reduced prematurity
PROLONG	International placebo-controlled confirmatory RCT; women with prior singleton spontaneous preterm birth	Weekly intramuscular 17-OHPC 250 mg vs. placebo	Preterm birth < 35 weeks and neonatal morbidity composite	No reduction in recurrent preterm birth or neonatal morbidity	Key reason for regulatory reassessment and withdrawal; illustrates historical inconsistency
Cuijpers et al.	Follow-up study of children exposed to progesterone for short cervix	Prenatal vaginal progesterone exposure	Two-year infant outcomes	No clear adverse developmental signal at approximately 2 years	Reassuring for early childhood, but insufficient for later neurodevelopmental outcomes
Simons et al.	Systematic review of randomized follow-up studies	Prenatal progesterone treatment	Child development, behavior, and health	No evidence of benefit or harm from 6 months to 8 years; limited first-trimester and later follow-up	Strongest synthesis of pediatric follow-up; highlights major evidence gaps
Su et al.	Retrospective observational study	Maternal progesterone exposure during pregnancy	Gesell developmental domains	Lower language and personal–social scores; other domains not significantly different	Hypothesis-generating; confounding by indication remains important
Murphy et al.	Long-term observational cohort with cancer registry linkage	In utero 17-OHPC exposure	Cancer risk in offspring	Possible increased cancer risk, especially with early exposure	Formulation-specific long-latency safety signal; not generalizable to natural progesterone

**Table 4:** Principal evidence gaps and future research priorities.

Evidence Gap	Current Limitation	Research Priority
Long-term follow-up	Most studies end in infancy or early childhood	Follow-up into school age, adolescence, and adulthood
Formulation heterogeneity	Natural and synthetic agents are often grouped together	Evaluate each formulation, route, dose, and exposure window separately
Comparator heterogeneity	Placebo trials, untreated cohorts, and observational studies are mixed	Predefine and report comparator/control structure
Confounding by indication	Maternal indication may influence offspring outcomes independently of treatment	Adjust for infertility, ART, miscarriage history, bleeding, cervical length, previous preterm birth, comorbidity, and prematurity
Historical practice changes	Indications, doses, formulations, and regulatory status changed over time	Use era-specific interpretation and harmonized treatment reporting
Placental evidence	Biologically plausible pathways are insufficiently studied	Add histology, biomarkers, transcriptomics, and epigenetic analyses
Sex-specific evidence	Male and female fetuses may differ in placental adaptation	Report fetal sex-stratified analyses
Population data	Rare or delayed outcomes require large cohorts	Use linked pregnancy medication and maternal-child registries

## Data Availability

No new data were created or analyzed in this study. Data sharing is not applicable to this article.
